# BDNF contributes to angiotensin II-mediated reductions in peak voltage-gated K^+^ current in cultured CATH.a cells

**DOI:** 10.14814/phy2.12598

**Published:** 2015-11-04

**Authors:** Bryan K Becker, Han-jun Wang, Changhai Tian, Irving H Zucker

**Affiliations:** Cellular and Integrative Physiology, University of Nebraska Medical CenterOmaha, Nebraska

**Keywords:** AT1R, p38 MAPK, SB-203580, TrkB

## Abstract

Increased central angiotensin II (Ang II) levels contribute to sympathoexcitation in cardiovascular disease states such as chronic heart failure and hypertension. One mechanism by which Ang II increases neuronal excitability is through a decrease in voltage-gated, rapidly inactivating K^+^ current (*I*_A_); however, little is known about how Ang II signaling results in reduced *I*_A_. Brain-derived neurotrophic factor (BDNF) has also been demonstrated to decrease *I*_A_ and has signaling components common to Ang II. Therefore, we hypothesized that Ang II-mediated suppression of voltage-gated K^+^ currents is due, in part, to BDNF signaling. Differentiated CATH.a, catecholaminergic cell line treated with BDNF for 2 h exhibited a reduced *I*_A_ in a manner similar to that of Ang II treatment as demonstrated by whole-cell patch-clamp analysis. Inhibiting BDNF signaling by pretreating neurons with an antibody against BDNF significantly attenuated the Ang II-induced reduction of *I*_A_. Inhibition of a common component of both BDNF and Ang II signaling, p38 MAPK, with SB-203580 attenuated the BDNF-mediated reductions in *I*_A_. These results implicate the involvement of BDNF signaling in Ang II-induced reductions of *I*_A_, which may cause increases in neuronal sensitivity and excitability. We therefore propose that BDNF may be a necessary component of the mechanism by which Ang II reduces *I*_A_ in CATH.a cells.

## Introduction

The development of chronic heart failure (CHF) and hypertension is often associated with an increase in sympathetic nervous system activity (Zucker et al. 2012). Although this increase serves a compensatory role during the early stages of CHF, over time, it further contributes to impairments in cardiovascular function and the development of associated complications such as renal failure (Zucker et al. 2012). Increased circulating levels of angiotensin II (Ang II) during the progression of CHF have been shown to contribute to sympathoexcitation (Guyenet 2006; Zucker et al. 2009). Elevated circulating Ang II may activate brainstem neurons in presympathetic control areas, such as the rostral ventrolateral medulla (RVLM), by initiating disruption of the blood–brain barrier (Biancardi et al. 2014) or stimulating circumventricular neurons such as in the subfornical organ (Zimmerman 2002; Zimmerman et al. 2004). Furthermore, increased activity of a local brain renin–angiotensin system in areas such as the RVLM may promote the development of increased sympathetic outflow. For instance, Gao et al. (2008) have shown an increase in Ang II Type 1 receptor (AT1R) expression in the RVLM of animals with experimental CHF. Therefore, therapies that interrupt sympathetic nervous system activity or inhibit Ang II signaling have been widely utilized to slow the development of CHF. However, it is not completely clear how Ang II increases the neuronal activity.

One way in which Ang II may increase sympathetic outflow is by increasing the sensitivity and excitability of presympathetic neurons through suppression of voltage-gated K^+^ channels that conduct the voltage-sensitive, rapidly inactivating current, *I*_A_. Acutely, Ang II inhibits *I*_A_ by increasing cellular levels of superoxide anion (Yin et al. 2010). In the RVLM of experimental models of CHF, there is reduced expression of the voltage-gated K^+^ channel protein (Kv)4.3, that has been demonstrated in the RVLM of experimental models of CHF (Gao et al. 2010). This protein contributes to the generation of *I*_A_ (Sonner and Stern 2007; Carrasquillo et al. 2012). In agreement with these findings, in vitro studies have demonstrated reduced *I*_A_ amplitude and Kv4.3 expression following 6-h treatment of catecholaminergic CATH.a cells with Ang II (Gao et al. 2010).

How Ang II elicits long-term reductions in Kv channel expression and *I*_A_ amplitude is not well understood, and whether other factors are involved has been incompletely investigated. In this regard, brain-derived neurotrophic factor (BDNF) signaling has been shown to modulate *I*_A_ in a manner similar to that of Ang II (Youssoufian and Walmsley 2007; Cao et al. 2010), and BDNF-induced increases in neuronal excitability have been extensively investigated (Huang and Reichardt 2003; Minichiello 2009). Furthermore, Ang II has been demonstrated to increase the expression of BDNF in neurons and other tissues (Chan et al. 2010; Szekeres et al. 2010). To the best of our knowledge, no studies have investigated the interaction of Ang II and BDNF in modulating *I*_A_ and neuronal excitability. Therefore, we hypothesized that the Ang II-mediated reduction in voltage-gated K^+^ currents is due, in part, to BDNF signaling. To test this hypothesis, we measured *I*_A_ in CATH.a cells treated with Ang II or BDNF. We also investigated the contribution of Ang II and BDNF on the p38 MAPK axis, which is an important component of both Ang II and BDNF signaling (Katoh-Semba et al. 2009; Xiao et al. 2013).

## Materials and Methods

### Chemicals

Ang II, losartan, and SB-203580 were purchased from Sigma–Aldrich (St. Louis, MO). Human recombinant BDNF, rabbit polyclonal antibody for BDNF (H-117), and mouse monoclonal alpha-tubulin antibody (10D8) were purchased from Santa Cruz Biotechnology Inc. (Santa Cruz, CA). All chemicals and compounds for electrophysiological solutions were purchased from Sigma–Aldrich unless otherwise stated.

### Cell culture

CATH.a cells were purchased from American Type Culture Collection (Manassas, VA), grown in RPMI 1640 medium supplemented with 8% horse serum, 4% fetal bovine serum, and 1% penicillin/streptomycin obtained from Gibco (Life Technologies, Grand Island, NY), and maintained in a humidified incubator at 37°C with 5% CO_2_. Cells were differentiated by incubating them in serum-free RPMI medium for 48–72 h as has been described previously (Qi et al. 1997; Mitra et al. 2010). Differentiated CATH.a cells were then treated with the designated agent dissolved in PBS and incubated in serum-free medium for the specified time period after which they were collected for western blot analysis or electrophysiology.

### Electrophysiology

Electrophysiological recordings were conducted similar to those previously reported from our laboratory and others (Gao et al. 2010; Yin et al. 2010). In brief, medium from the polystyrene dish with differentiated CATH.a cells was aspirated, the cells were washed with PBS, and extracellular electrophysiology solution was added to the dish. The extracellular solution contained (in mmol/L): 140 NaCl, 5.4 KCl, 0.5 MgCl_2_, 5.5 HEPES, 11 glucose, and 10 sucrose. It was buffered to pH of 7.4 with NaOH and 0.5 *μ*mol/L tetrodotoxin was added in order to block TTX-sensitive, voltage-gated Na^+^ channels. Patch pipettes were pulled using a P-97 Flaming/Brown micropipette puller (Sutter Instruments, Novato, CA), fire polished to a final resistance of 2-4 MΩ, and filled with solution containing (in mmol/L): 105 potassium aspartate, 20 KCl, 10 EGTA, 5 Mg-ATP, 10 HEPES, and 25 glucose, adjusted to pH 7.2 with KOH. K^+^ currents were recorded by an Axopatch 200B amplifier and digitized using a Digidata 1440A interface (Molecular Devices, Sunnydale, CA). Data collection and analysis were done using pCLAMP 10 software (Molecular Devices). Whole-cell membrane capacitance was determined by canceling the capacitive current evoked by a 10-mV voltage step. Currents were not leak subtracted, and signals were filtered at 5 kHz and sampled at 10 kHz. Cells were held at -60 mV or -80 mV, and 400-ms duration voltage steps were applied in 10-mV increments to +80 or +110 mV.

Peak current amplitude (pA) representing *I*_A_, time to peak current (ms), time of activation (*τ*_act_; ms) or decay (*τ*_decay_; ms), and the maximum slope during activation or decay were measured at each voltage step after the total trace had been normalized by the whole-cell membrane capacitance using analysis functions in pCLAMP software.

### Western blotting

After treatment, CATH.a cells were scraped from polystyrene culture dishes and immediately placed in ice-cold RIPA buffer supplemented with protease inhibitor cocktail (P8340, Sigma–Aldrich) and phosphatase inhibitor cocktail (P5726, Sigma–Aldrich). Cells were homogenized by sonication and total protein concentration was estimated by a Pierce BCA protein assay kit (Rockford, IL). A total of 20 *μ*g (approximately 20 *μ*L) of protein was boiled for 5 min in an equal volume of 4% SDS sample buffer and was loaded into a 7.5 or 12% SDS-PAGE gel, and ran at 100 V for approximately 1 h on a Bio-Rad mini gel electrophoresis apparatus (Hercules, CA). The protein was then transferred to a nitrocellulose membrane (Li-Cor, Lincoln, NE) at 50 V for 90 min. Membranes were then blocked in a 1:1 solution of Li-Cor blocking solution (Lincoln, NE) and PBS for 1 h. Membranes were then incubated overnight at 4°C in PBS with primary antibodies to BDNF (1:2000) and alpha-tubulin (1:5000), and incubated with Li-Cor secondary infrared-labeled antibodies (IRDye 680LT 926-68022 at 1:10,000 and IRDye 800CW 926-32214 at 1:5000) for 1 h at room temperature in PBS with 1% SDS. Bands were visualized using a Li-Cor Odyssey system and analyzed using Li-Cor Odyssey software.

### Statistics

All data are expressed as mean ± SEM. One-way analysis of variance was used to compare group differences between western blot data with Dunn’s post-hoc analysis. A repeated measures one-way analysis of variance was used to determine the treatment interactions in electrophysiological IV measurements. SigmaPlot 11.0 (Systat Software Inc., San Jose, CA) and SPSS (IBM, Armonk, NY) were used to complete statistical analysis. A *P* value of <0.05 was used to determine statistical significance.

## Results

### Ang II increases BDNF expression

**Figure 1:**
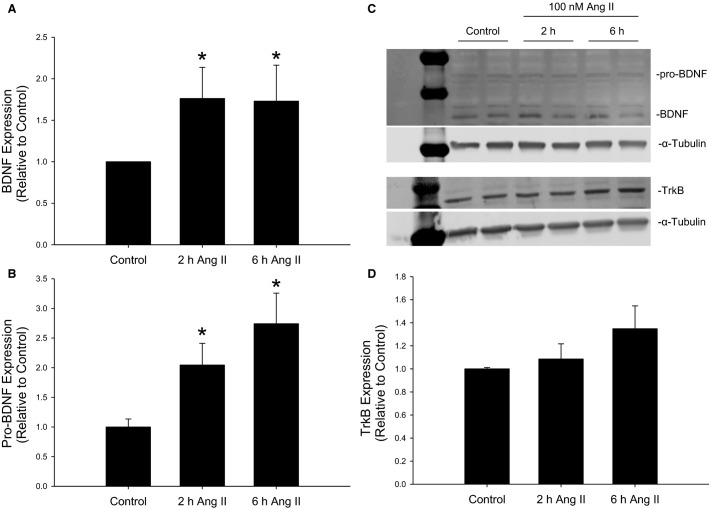
BDNF and TrkB expression in CATH.a cells following Ang II treatment. Representative blots (C) and relative expression levels of BDNF (A), pro-BDNF (B), and TrkB (D) following treatment of CATH.a cells with 100 nmol/L Ang II for 2 or 6 h. *P < 0.05 versus Control treatment, *n* = 5–8/group.

Following treatment of CATH.a cells with 100 nmol/L Ang II for 2 or 6 h, both pro-BDNF and BDNF expression increased relative to nontreated controls as measured by western blot (Fig.1A, B). We failed to observe an increase in the relative expression levels of TrkB following Ang II treatment at either 2 or 6 h (Fig.1D).

### BDNF reduces *I*_A_

**Figure 2:**
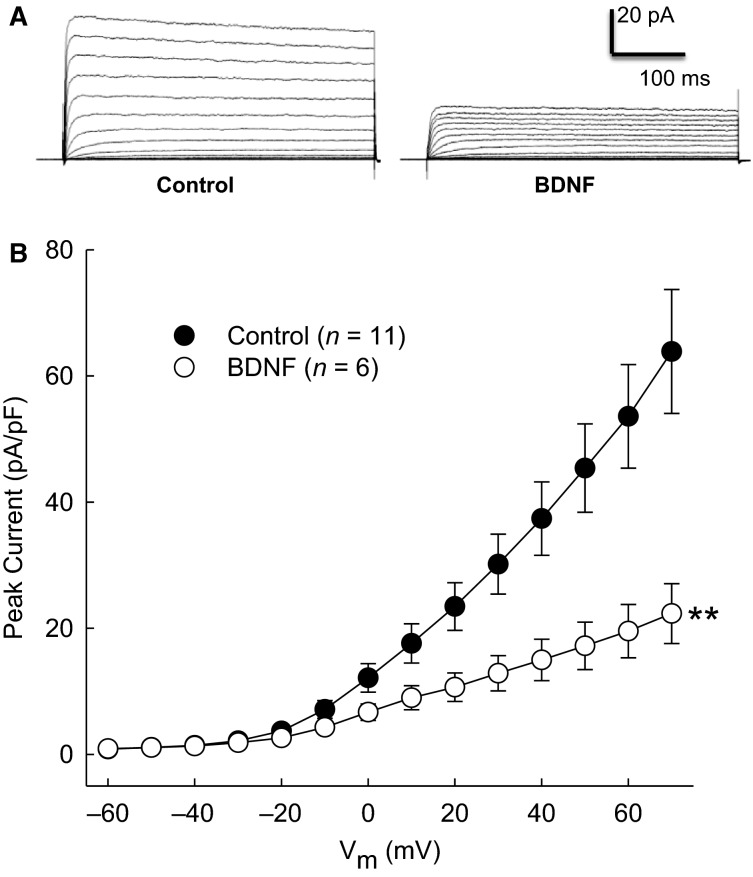
Effect of BDNF on *I*_A_. Representative traces (A) and mean I–V plots of peak K^+^ current density (B) in CATH.a neurons treated with 50 ng/mL BDNF for 2 h. *P < 0.05 interaction between groups as measured by RM-ANOVA.

To investigate whether BDNF affects *I*_A_ in CATH.a cells, patch-clamp experiments were performed. Previous reports have demonstrated reductions in voltage-gated K^+^ currents following 50 ng/mL of BDNF after 2–4 h (Cao et al. 2010, 2012). Treatment of neurons with 50 ng/mL of BDNF for 2 h reduced mean *I*_A_ by 65% during a voltage step to +70 mV (Fig.2). Because this effect was similar to the previously reported reduction of *I*_A_ due to Ang II treatment (Gao et al. 2010), and because Ang II has also been shown to rapidly suppress voltage-gated K^+^ current (Yin et al. 2010), we explored whether an acute treatment with BDNF would produce a similar effect to that of acute application of Ang II. However, peak current was not altered after superfusion of CATH.a cells with 50 ng/mL BDNF for 10 min (44.1 ± 7.5 pA/pF before BDNF superfusion vs. 40.4 ± 6.7 pA/pF 10 min after BDNF at +70 mV voltage step, *n* = 5/group, *P *=* *0.96 between groups).

### BDNF is involved in the Ang II-induced reduction of *I*_A_

**Figure 3:**
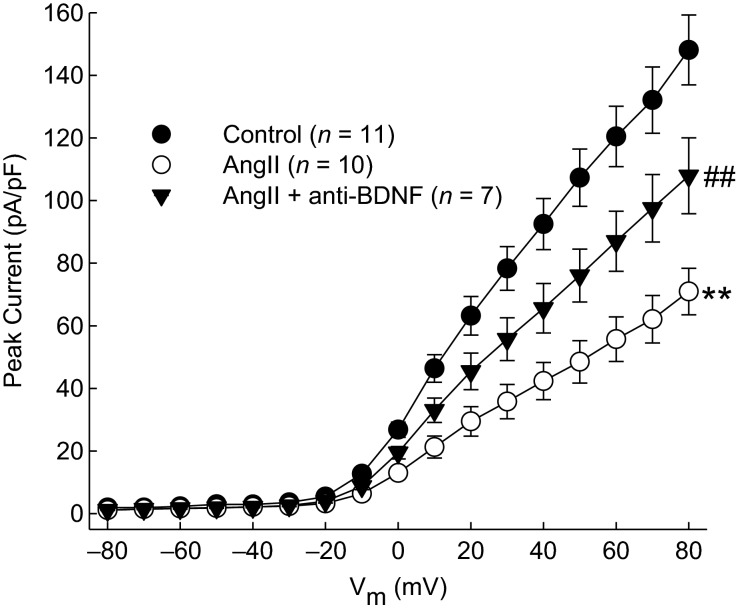
Effect of inhibiting BDNF on Ang II-induced suppression of *I*_A_. Mean I–V plots of peak K^+^ current density of CATH.a neurons incubated with 100 nmol/L Ang II for 6 h or pretreated with 50 ng/mL anti-BDNF antibody for 30 min prior to Ang II. **P < 0.01 group interaction versus Control and ##*P* < 0.01 versus Ang II group interaction as measured by RM-ANOVA.

Ang II has been demonstrated to reduce *I*_A_ (Gao et al. 2010); however, the involvement of other factors in this phenomenon has not been elucidated. Based upon the ability of BDNF to reduce *I*_A_, we investigated the involvement of BDNF in the Ang II-induced reduction of *I*_A_. Inhibition of endogenous BDNF signaling by pretreatment with an anti-BDNF antibody attenuated the reduction in peak current following incubation with Ang II (Fig.3). In order to determine if anti-BDNF antibody had any independent effects on K^+^ current, CATH.a cells were incubated with anti-BDNF antibody alone. Peak current was not altered by incubation of neurons with anti-BDNF antibody alone relative to control (116.0 ± 10.7 pA/pF at +80 mV voltage step, *n* = 7, *P *=* *0.74 between groups).

Because BDNF or Ang II can independently reduce *I*_A_, and because BDNF signaling is involved in the mediation of the Ang II-induced reduction in *I*_A_, we investigated whether Ang II signaling is involved in the BDNF-induced suppression of *I*_A_. Cells were pretreated with 100 nmol/L losartan, an AT1R blocker, for 30 min prior to 50 ng/mL BDNF incubation for 2 h. *I*_A_ was not altered in losartan-treated neurons (82.5 ± 13.1 pA/pF at +80 mV voltage step, *n* = 7) relative to BDNF treatment alone (82.4 ± 16.8 pA/pF, *n* = 6, *P *= 0.90 between groups).

### Involvement of p38 MAPK in the BDNF-induced reduction of *I*_A_

**Figure 4:**
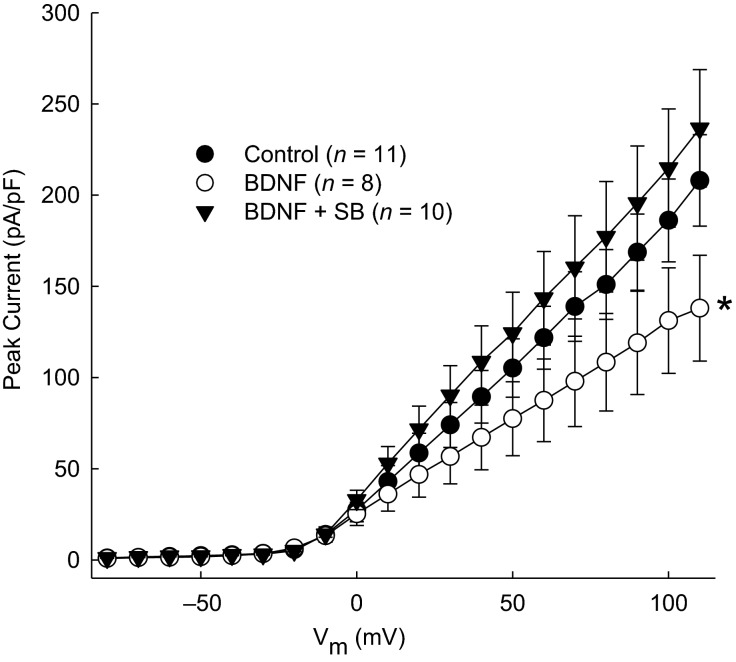
Effect of inhibition of MAPK on BDNF-induced reduction of *I*_A_. Mean I–V plots of peak K^+^ current density in CATH.a neurons incubated with 50 ng/mL BDNF for 2 h or pretreated with 100 nmol/L SB-203580 (SB) for 30 min prior to BDNF. *P < 0.05 group interaction between BDNF and SB+BDNF as measured by RM-ANOVA.

Previous results have demonstrated the involvement of p38 MAPK in Ang II-mediated reductions in *I*_A_ and downregulation of Kv4.3 protein (Gao et al. 2010). To determine if p38 MAPK is involved in the BDNF-induced reduction of *I*_A_, patch-clamp experiments were performed after treating CATH.a cells for 2 h with 50 ng/mL with or without pretreatment of the p38 MAPK inhibitor SB-203580 (100 nmol/L) for 30 min. Inhibiting p38 MAPK completely prevented the reduction in *I*_A_ following BDNF (Fig.4).

Time to peak current was measured following 50 ng/mL BDNF treatment for 2 h with or without 30-min pretreatment with 100 nmol/L SB-203580. Time to peak current during the voltage step to +80 mV was not changed following BDNF treatment (66.8 ± 21.9 ms, *P *=* *0.4, *n* = 8) relative to control (46.9 ± 17.4 ms, *n* = 8). Likewise, time to peak current was not changed by BDNF after cells were pretreated with SB-203580 (41.4 ± 9.0 ms, *P *=* *0.79, *n* = 10) relative to BDNF.

Next, to evaluate whether BDNF affects kinetic properties of *I*_A_, various time-related parameters were calculated following incubation of CATH.a cells with 50 ng/mL BDNF for 2 h with or without 30-min pretreatment of 100 nmol/L SB-203580. BDNF treatment increased mean *τ*_act_ by 52 ms relative to control (Table1). The *τ*_act_ was not attenuated by pretreatment with SB-203580 (*P* = 0.97 relative to BDNF alone). The other kinetic parameters measured (*τ*_decay_, maximum rise slope, and maximum decay slope) were not affected by BDNF or SB-203580 (Table1).

**Table 1:** Effects of BDNF with or without p38 MAPK inhibition on kinetic parameters of *I*_A_

Treatment	*τ*_act_ (ms)	*P*	*τ*_decay_ (ms)	*P*	Max Rise Slope (pA/ms)	*P*	Max Decay Slope (pA/ms)	*P*	*N*
Control	5.5 ± 4.2		48.5 ± 16.3		31.9 ± 7.1		−31.0 ± 6.3		8
BDNF	58.0 ± 15.3	0.001	17.0 ± 5.0	0.16	25.8 ± 3.9	0.96	−23.3 ± 3.5	0.33	8
SB + BDNF	55.3 ± 18.0	0.04	32.4 ± 9.6	0.65	39.6 ± 3.6	0.09	−35.6 ± 3.5	0.53	10

*Note*: *P* values are relative to Control group. *τ* = time of activation (act) or decay.

## Discussion

The major findings of this study are that BDNF reduces peak *I*_A_ and that the Ang II-induced decrease in *I*_A_ in CATH.a cells is attenuated by inhibiting the action of BDNF (Fig.3), and that p38 MAPK is involved in the signaling of BDNF-induced reductions in *I*_A_ (Fig.4). These results suggest that BDNF and p38 MAPK may be key mediators involved in the reduction of *I*_A_ due to Ang II. Previous reports have demonstrated reduction in *I*_A_ following 100 nmol/L Ang II treatment for 6 h (Gao et al. 2010), similar to our present findings (Fig.3). However, little is known about the signaling cascades involved in this Ang II-mediated change in electrophysiological phenotype. Here, we demonstrate the upregulation of BDNF protein following Ang II treatment and the involvement of BDNF in the Ang II-induced reduction of *I*_A_.

Ang II is known to have immediate effects on K^+^ currents and neuronal firing through signaling by reactive oxygen species. Specifically, Ang II elicits an increase in intracellular superoxide anion that inhibits peak and steady-state K^+^ currents within 10 min (Yin et al. 2010). Our results suggest that BDNF may not be involved in acute modulation of K^+^ currents because no changes to peak K^+^ current were observed following 10-min superfusion of BDNF. Thus, Ang II may have multiple modes of modulating K^+^ currents: acutely, by generation of reactive oxygen species; and in the long term, through BDNF signaling. Furthermore, these results suggest that the reduction of *I*_A_ following treatment with Ang II or BDNF for several hours is likely due to a decrease in the expression of channels responsible for *I*_A_ such as Kv4.2 or Kv4.3 and not due to direct inhibition of K^+^ channel activity. Although BDNF increased *τ*_act_, other kinetic parameters of peak K^+^ current remained unchanged, indicating that the main action of BDNF on suppressing *I*_A_ are likely through reducing the total expression of Kv4.3, which correlates well with our previous results demonstrating reductions in Kv4.3 expression following Ang II treatment (Gao et al. 2010).

Ang II has been shown to act as a neurotransmitter that depolarizes neurons and increases excitability (Oz and Renaud 2002; Latchford and Ferguson 2005; Zaika et al. 2006), and BDNF is released in response to neuronal activity to facilitate the development of long-term potentiation (Huang and Reichardt 2003; Nagappan and Lu 2005; Minichiello 2009). These events raise the possibly that the development of sympathoexcitation in CHF or some forms of hypertension could be due to the interplay between Ang II-elicited increases in neuronal activity in brainstem nuclei, such as the RVLM, and aberrant development of long-term potentiation through BDNF. Further investigation is needed to determine if Ang II causes an increase in BDNF activity through signaling cascades or if BDNF activity is increased due to increased neuronal activity stimulated by Ang II.

A recent study by Erdos et al. (2015) demonstrated that overexpression of BDNF in neurons of the paraventricular nucleus was sufficient to raise blood pressure, heart rate, and markers of sympathetic tone, implicating the ability of BDNF to modulate presympathetic neuronal pathways and increase sympatho-excitation. Interestingly, these effects were attenuated by ICV administration of the AT1R blocker losartan suggesting the critical role of the Ang II signaling in the mechanism of BDNF signaling. This study along with our current data suggests a possible convergent signaling and bidirectional interaction of the Ang II and BDNF pathways. It remains to be seen if the convergence of these signaling pathways is involved in mediating the sympathoexcitatory conditions seen during disease states such as heart failure and hypertension.

It has been shown that the inhibition of p38 MAPK with SB-203580 can attenuate the reduction in Kv4.3 mRNA following Ang II treatment (Gao et al. 2010). Here, we demonstrate that SB-203580 can prevent the reduction in *I*_A_ following treatment with BDNF. These observations, along with the involvement of BDNF in Ang II-induced reductions in *I*_A_, suggest that p38 MAPK plays a role in the convergence of BDNF and Ang II signaling.

A potential limitation of the current study is the use of an anti-BDNF antibody to inhibit BDNF signaling through its receptor tyrosine kinase (TrkB). Other potential methods to block BDNF signaling through TrkB include pharmacological inhibition using drugs such as K252a. Although often cited as a specific inhibitor of TrkB, K252a can also inhibit the action of a number of tyrosine kinases including p38 MAPK (Martin et al. n.d[Bibr b16];.). As we were interested in the activity of p38 MAPK in this study, this precluded the use of K252a. Use of anti-BDNF antibody to prevent BDNF-TrkB interaction has demonstrated to be an effective inhibitor of BDNF–TrkB signaling (Cao et al. 2012).

The CATH.a cell model has been extensively used in investigations of Ang II signaling and K^+^ current modulation (Gao et al. 2010; Yin et al. 2010; Yang et al. 2011; Haack et al. 2012; Xiao et al. 2013). This cell line fully expresses the necessary proteins for Ang II signaling and expresses a number of K^+^ channels including Kv4.3. Therefore, it is appropriate for investigation of the effects of Ang II on K^+^ currents. In our hands, we are unable to elicit action potentials from CATH.a cells, which limits the direct application of these results to sympathoexcitation in which we were unable to observe changes in neuronal sensitivity and action potential frequency. However, previous studies using acutely dissociated neonatal neurons have demonstrated similar effects of Ang II on K^+^ currents (Kang et al. 1992, 1993; Pan et al. 2001). More robust electrophysiological experimentation such as brain slice measurements or acutely dissociated neurons from animal models of CHF is needed to confirm these current findings and previous studies in integrative physiological systems. Moreover, although our data demonstrate important molecular and signaling interactions in modulating K^+^ currents in vitro, further investigations are needed to observe the impact of these findings to whole-animal physiology during the progression of CHF and other Ang II-mediated disease states associated with increased sympathetic activity. In particular, the interaction of BDNF and Ang II mediating sympathoexcitation in brainstem nuclei during CHF and other sympathoexcitatory states need to be explored.

These findings indicate the involvement of BDNF signaling in Ang II-mediated reductions of *I*_A_, and suggest the convergence of BDNF and Ang II signaling on p38 MAPK. These data provide new insight into the mechanisms responsible for altered intrinsic neuronal excitability in diseases characterized by sympathoexcitation such as CHF and hypertension. Further investigation into the specific channels and neuronal populations affected by BDNF and Ang II in the intact animal will be beneficial in further understanding the basic physiology and treatment possibilities for these prevalent diseases.
